# The sociology of cancer: a decade of research

**DOI:** 10.1111/1467-9566.12662

**Published:** 2018-02-15

**Authors:** Anne Kerr, Emily Ross, Gwen Jacques, Sarah Cunningham‐Burley

**Affiliations:** ^1^ School of Sociology and Social Policy University of Leeds UK; ^2^ The Usher Institute Edinburgh Medical School University of Edinburgh UK

**Keywords:** biomedicine, cancer, identity, risk, science and technology studies (STS)

## Abstract

Biomedicine is often presented as the driving force behind improvements in cancer care, with genomics the latest innovation poised to change the meaning, diagnosis, treatment, prevention and lived experience of cancer. Reviewing sociological analyses of a diversity of patient and practitioner experiences and accounts of cancer during the last decade (2007–17), we explore the experiences of, approaches to and understandings of cancer in this period. We identify three key areas of focus: (i) cancer patient experiences and identities; (ii) cancer risk and responsibilities and (iii) bioclinical collectives. We explore these sociological studies of societal and biomedical developments and how sociologists have sought to influence developments in cancer identities, care and research. We end by suggesting that we extend our understanding of innovations in the fields of cancer research to take better account of these wider social and cultural innovations, together with patients, activists' and sociologists' contributions therein.

## Introduction

As populations age and processes of biomedicalisation expand, cancer has become a major focus of biomedical research and services across the world. In the UK, as a proportion of medical research spending for four common conditions (cancer, coronary heart disease, dementia, and stroke), cancer research takes the highest proportion, with around 64 per cent of funding from governmental and charity organisations going to cancer in 2012 (Luengo‐Fernandez *et al*. 2015). Funds allocated to the United States National Cancer Institute (NCI), part of the national medical research agency the National Institutes of Health (NIH), totalled $5.2 billion for 2016. This comprised over 16 per cent of the US congress budget for all NIH activities. Recent estimations for global spending on cancer medications suggest a figure of $100 billion (Leonard 2015).

The diagnosis, treatment, and care for patients affected by cancer are also a major focus within hospital and primary care settings. The Nuffield Trust, a UK independent health charity, estimates that cancer was the third largest disease category of UK spending in 2013–14 (Nuffield Trust 2015). The incidence of cancer is also rising: recent estimates suggest that 1 in 2 people in the UK will experience cancer during their lifetime (Ahmad *et al*. 2015, Cancer Research UK 2015). The affordability of cancer treatments is also a major challenge for private individuals as well as state funded health services such as the UK NHS. At the same time, cancer is never far from the public eye, dominating the landscape of charity fundraising. Cancer charities turn over millions of pounds annually (for example, Cancer Research UK had a revenue of £537 million in 2013 (Cancer Research UK 2014)). High profile cancers like breast cancer have also become associated with strong discourses of patient and citizen empowerment (Bell 2014, Gibson *et al*. 2015). Cancer is also at the forefront of efforts to enhance diagnosis and prognosis via targeted treatments and therapies, with participation in research, especially clinical trials, becoming a much more routine part of the cancer experience (Keating and Cambrosio 2007, 2012).

During this period cancer has also been a major focus of sociological inquiry, featuring prominently in the pages of this and other journals in the field, including contributions from medical sociologists who have had direct experience of cancer (e.g. Blaxter 2009, Horlick‐Jones 2011). In the period 1995–present, arguably a period where the field of medical sociology has consolidated and flourished (Bloom 2002; Seale 2008), there were 244 articles published in the journal *Social Science and Medicine* alone that mentioned ‘cancer’ in the title: a high level compared to articles mentioning the other ‘big killers’ in the Western world (e.g. 37 article titles mentioned ‘heart disease’ and 16 articles that mentioned ‘stroke’). In addition to exploring patient experience, medical sociologists, alongside colleagues from science and technology studies (STS) and medical anthropology, have closely followed developments in cancer diagnostics, screening and treatment, including with respect to approaches towards hereditary cancers where genetic and more recently genomic research and information have been incorporated into clinical practice (Aasen and Skolbekken 2014, Hallowell *et al*. 2009, Löwy and Gaudillière 2008, Prior 2007).

In this article, we review the significant corpus of sociological literature on cancer over the past decade to explore the insights it has provided about cancer research, services and experiences in this period, tracing social as well as biomedical developments and their impact on patients and families. We identify the dominant themes and issues in this work and consider the way in which it mirrors and challenges wider societal and biomedical developments. We consider the role sociological research can play in effecting changes in the research, services and communities under consideration, and inform the efforts of activists, practitioners, scientists and patients to improve care for those affected by cancer.

## Methodology

Our aim is to provide an up to date review and critical analysis of medical sociological knowledge about cancer and the experience thereof. We have taken a narrative approach while seeking to be pragmatically inclusive. While the authors are familiar with a range of relevant literature, to be comprehensive we conducted a search of the database Sociological Abstracts, covering the period 2007–17. Sociological Abstracts gives ample coverage of the international literature of sociology and related disciplines. There are, of course, limits to any database search; we found a few articles that were missed from the search due to either our search criteria or the databases own functionality; these have been included as part of the review. The database does not retrieve books and there are several relevant to the sociological developments discussed in this article (e.g. Ehrenreich 2009, Jain 2013). These have been included. Some articles published prior to 2007 have also been referenced; such prior work has informed the contemporary field and, hence, the structure of the review.

For a topic as large as cancer, we needed to provide some narrowing of focus. We concentrated our review on a decade of research on adult cancers and cancer in mainstream healthcare, excluding articles which considered a range of diseases not just cancer, articles about childhood or adolescent cancer, or complementary and alternative therapies. This review concentrates on sociological literature, while recognising that disciplinary boundaries are fluid. We searched for English‐language articles including ‘cancer’ or ‘oncolog*’ in the abstract or title (465 in total); we then read all of the abstracts and excluded articles from psychology, epidemiology, demography, evaluations of or about service delivery and development. We also excluded the few review articles we identified.

Three authors separately applied the exclusion criteria described above to the first 100 identified article abstracts, and resolved disagreements after reading the full article. Using the criteria refined during the process of excluding articles from these initial 100, the remaining 365 abstracts were divided between the first and last author, with the second author reading the full set. Exclusion criteria were applied by each author separately, and discrepancies with the second author's exclusion choices were discussed and resolved. Several articles were singled out to be read in full during this process. Most articles reported on primary research, most frequently of a qualitative nature.

In total, 227 articles from the search were taken forward for the review. These articles are presented in a separate bibliography in the Appendix. Books and additional articles identified outside the database search are referenced at the end of this review. Through discussions between authors during the reviewing and reading, three key themes were identified: experiences of living with and beyond cancer, cancer risk and responsibility, and bioclinical collectives. Articles were then assigned to each of these themes. Where articles were positioned within multiple themes, discrepancies were resolved via discussion between these three authors. We now discuss each theme in turn, providing a broad narrative of how the topic has been treated and developed.

## Living with and beyond cancer

Sociology has a long tradition of qualitative research into the experiences of illness, from the early work in the social constructivist tradition (Blaxter and Paterson 1982), to recent studies of chronic illness and the body from a more Foucauldian perspective (Armstrong 2003, Gibson *et al*. 2015). This work is notable for its emphasis on the lived experience of disease, not just in terms of its physical manifestations and tribulations, but also with respect to identity work and caring practices. These are also important themes in the sociology of cancer.

Of the 227 articles we identified, the largest proportion (101) concerned living with and beyond cancer. These studies drew largely on qualitative research into people's experiences of cancer, from diagnosis to treatment and support. Articles tended to focus on experiences of particular cancers (65) with a smaller number considering a range of cancers (36). Of those articles about living with and beyond a particular cancer, the majority (47) focused on women's experiences of breast and/or gynaecological cancers (33 breast cancer and 14 gynaecological), with six considering prostate cancer in men. Very few considered other cancers, such as bone (Parsons *et al*. 2008) and blood cancer (Schaepe 2011). The majority of studies focused on white Europeans, although some did focus on Black and Minority Ethnic (BME) or religious minorities, whether in Europe/North America or Australia or in other countries. These included research with members of the Maori population (Seneviratne *et al*. 2015), Bahraini women (Jassim and Whitford 2014), African Americans and Caribbean individuals living in the United States (Bache *et al*. 2012, DiIorio *et al*. 2011), and immigrant women's experiences of survivorship (Burke *et al*. 2012). Often exploration of these aspects of identity intersected with consideration of socioeconomic inequality (Mulemi 2008).

These articles covered the following areas: identity work, biographical disruption, lived experiences, lay beliefs, stigma and anomie (35); support, care and coping, survivorship, quality of life, dying, palliation, faith (43); patient‐caregiver engagement, trust, decision‐making, delayed diagnosis/treatment, barriers to care, surveillance (13); and, gender (10). We have grouped these around two broader themes – identity and subjectivity and cancer and care work.

### Identity and subjectivity

Research on identity (35) was primarily concerned with how cancer reconfigures identities, tracing the positive and negative effects of these changes. This included several articles considering ‘biographical disruption’ (Bury 1982), including how advanced stage cancer limits everyday existence and requires a re‐evaluation of one's future (Brown and de Graaf 2013) and selfhood (Hubbard *et al*. 2010, Reeve *et al*. 2010, Sinding and Wiernikowski 2008). These studies explored how participants lived with and beyond cancer through an emphasis on getting by and making the best of their situation. Some authors utilised the idea of biographical disruption to show how cancer ‘threaten(s) biographical trajectory and self‐identity forever’ (Balmer *et al*. 2015: 451), creating a sense of liminality and ambiguous survivorship (Roberts and Clarke 2009; Trusson *et al*. 2016), continuing long after treatment ended (Andersen *et al*. 2008). Others suggested that biographical disruption is not necessarily a common experience for people living with and beyond cancer, and can in fact be ‘foreclosed’ by social conventions (Sinding and Wiernikowski 2008). Reeve *et al*. (2010) drew on these insights to suggest that medical sociology needs to focus on understanding how practitioners can help alleviate disruption to support patients.

Others explored Frank's narrative‐based analysis, in particular his notion of the restitution narrative associated with biomedical or modernist approaches to conquering illness (Frank in Coll‐Planas and Visa 2016, Sparkes *et al*. 2012) and the quest narrative, based around a rejection of biomedicine and a search for alternative meanings in the post‐modern age. Once again, these studies challenge rather than reassert the theoretical framework under exploration. For example, Coll‐Planas and Visa (2016) did not find the dichotomy between biomedical, restitution (modernist) and challenging quest (post‐modern) narratives in their analysis of a series of breast cancer blogs, but instead identified a series of intertwined narratives of survival, gratitude, relationality, positivity and personal growth (see also Hansen and Tjornhoj‐Thomsen 2008).

The wider set of articles concerned with meanings and experiences of cancer diagnosis, treatment and the spread or recurrence of cancer also focused on how people living with the effects of cancer surgery realigned personhood (Ramirez *et al*. 2014), regained wholeness (Crompvoets 2012) and in some cases integrated test results into ‘technoscientific illness identities’ (Bell and Kazanjian 2011, Jordens *et al*. 2010, Sulik 2009). This literature challenged dominant narratives of fighting cancer, and depictions of cancer as prompting personal growth and positivity (Solbraekke and Lorem 2016, Willig 2011), echoing popular criticism of these tropes (e.g. Ehrenreich 2009). For example Cobb and Starr (2012) explored transforming identities in their research with breast cancer patients and the ‘makeover metaphor’. They describe disconnections between women's experiences of reconstructive surgery and the post‐feminist commodification of breast cancer in the ‘make‐over’ metaphor, arguing this ‘obscures the pain and complications of the disease and its treatments in an attempt to deny the body's limits’ (Cobb and Starr 2012: 98, see also DeShazer 2012).

### Cancer and care work

There were other critical challenges to dominant ways of thinking about living with and beyond cancer in the articles reviewed. Focusing on the disruptive effects of cancer treatment and recovery on identities in relation to work (Moffatt and Noble 2015, Parsons *et al*. 2008), several authors explored the various kinds of work involved in being ill and recovering, and advocated changes in clinical practice and welfare policy to reflect these activities. For example, Parsons *et al*. (2008) considered the different kinds of illness, identity and vocational work that bone‐cancer patients engage in through the course of their treatment and recovery, and called for an appreciation of these activities in support for patients returning to work. This included the need for clinicians to ‘listen differently to patients’ accounts (recognising their complex, nuanced nature) and ask better questions, tailored to individual circumstances’ (Parsons *et al*. 2008: 1834). Moffat and Noble (2015) also explored the stigma of welfare and called for a rights‐based approach to welfare claimants affected by cancer. This challenge to prevailing approaches to cancer care was also evident in research focusing on alienation. Building from the work of Stacey (1997), Blaxter (2009) drew on her own experiences of cancer to highlight the alienation she experienced as practitioners and protocols translated medical images into records and decisions without her involvement. Horlick‐Jones (2011) explored his fears of recurrence, considering how these feelings impacted on experiences of embodiment and emotional wellbeing.

Articles on care and coping were primarily focused on improving psychosocial relations and caring arrangements. Several articles considered the role of support groups, both in person and online (Bell 2009, Stephen *et al*. 2014, Vilhauer 2009), and a range of articles discussed spirituality (10). Many of these authors sought to understand the experiences of people of faith and different cultural contexts in an effort to improve care and coping (Coleman‐Brueckheimer *et al*. 2009, de Graaff *et al*. 2012, Koffman *et al*. 2008, Okamoto 2008). For example, Koffman's study found that Black Caribbean patients' faith was more important in their accounts of their cancer experiences than that of White British patients, and called for care‐providers to engage with spiritual beliefs among their Black Caribbean patients in discussing treatment decisions. Other articles addressed coping and communication strategies among cancer patients and carers (Donovan‐Kicken *et al*. 2011, Olson 2011), trust‐building (Katzman 2013), and positive relationships in families (see Wiggs 2011 on mother daugher relationships). Towsley *et al*. (2007) focused on how cancer survivors ‘learned to live’ with their experiences as a model for support services. Others discussed how to support patients in recognising, reformulating and actualising goals in the months prior to death (Nissim *et al*. 2012). In one of the few quantitative studies in the literature, Thompson *et al*. (2016) considered the influence of neighbourhood on experiences of early stage breast cancer patients, and advocated neighbourhood level interventions to improve levels of support.

The importance of reconceptualising care as an ‘embodied moral practice’ (Chattoo and Ahmad 2008), and as a temporal (Olson 2011) and intersubjective experience (Ussher *et al*. 2011) was an important theme in the qualitative articles on cancer care. This included research highlighting the need to consider multiple emotions, relationships and identities, rather than focusing on particular social categories of difference, such as ethnicity, when understanding caring experiences (Chattoo and Ahmad 2008). Wainer *et al*. (2012) also highlight that different levels of support may be required at different stages of experiences of cancer, with women they interviewed feeling particularly vulnerable in the post‐operative period. Articles also challenged taken‐for‐granted assumptions about the universal benefits of particular approaches to care. This included self‐care, as seen in Youll and Meekosha's (2013) discussion of ‘positive thinking’ as a ‘technology of the self’ (Rose in Youll and Meekosha 2013), and hope metrics (Brown 2015). Other critical interventions challenged common tropes such as ‘hero narratives’ (Sandaunet 2008), highlighted experiences of alienation in online support groups, or drew on cancer in artworks (Radley and Bell 2007) to explore how collective critique might be mobilised as part of ‘strategies for survival’ (Radley and Bell 2007: 386). A small but significant collection of articles considered the ways in which gender and sexuality shaped experiences of cancer and treatment (Blank 2008, Hordern and Street 2007, Manderson and Stirling 2007, Robbins *et al*. 2014, Ramirez *et al*. 2009, Seale and Charteris‐Black 2008, Wenger and Oliffe 2014), and the gendered nature of conventions and care (Bottorff *et al*. 2008, Ehlers 2012, Hansen 2007, Mroz *et al*. 2011, Sulik 2007a, 2007b). These articles challenged dominant tropes, for example those mobilised in breast cancer image and education programmes (Kendrick 2008, Phillips 2009).

Articles on patient‐caregiver interactions and service uptake were primarily focused on understanding and improving or supporting patients' understandings of risk and responsibility (Heyman *et al*. 2012, Mendick *et al*. 2010, Sinding *et al*. 2010) and their interactions with care‐givers, stressing the social processes that shape decision‐making, disclosure and interpretation of results (Gross 2012, Johansen *et al*. 2012, 2014, Sarradon‐Eck *et al*. 2012; Stacey *et al*. 2009). These included decision‐making beyond treatment, fertility preservation (Quinn *et al*. 2008) and participation in early‐stage trials (Jansen 2014). This literature included more applied, quantitative research, for example on the causes of inequalities in delayed diagnosis (Seneviratne *et al*. 2015) or barriers to screening uptake (Tsu and Levin 2008). Some qualitative work foregrounded relationality in a similar vein to some of the articles on care and identity. Here researchers emphasised and explored the situational and contextual factors which shaped patients' perceptions and experiences, such as ‘lay’ health beliefs (Drew 2011, James *et al*. 2011, van Schalkwyk *et al*. 2008). A particular focus within these articles was on how meanings are constructed via interaction. For example, Brown (2009) explored how trust is formed via inter‐personal communication, stressing the importance of understanding and respecting the rationality of lay decision‐making as an outcome of these processes (see also Brown *et al*. 2011). Others highlighted the new kinds of responsibilities patients acquire in a culture of healthcare which emphasises their involvement, calling for more attention to how these processes might exacerbate inequalities among patients (Sinding *et al*. 2012).

Together these sociological studies of living with and beyond cancer have documented and facilitated the social practices of ‘telling ones story’ of cancer, contributing to the rise of a ‘therapeutic culture’ of self‐improvement and care in cancer services and support. At the same time, these studies have traced patients' resistance to normative notions of survivorship and coping, especially gender norms. A range of studies have explored how individuals living with and beyond cancer experience care and its deficits, with a view to making encounters and experiences more sensitive and appropriate to their needs. Through this research sociologists have been actively involved with and therefore played a role in stimulating criticism of hegemonic ideas about how cancer survivors feel or how they should act, and in so doing have further exposed the risks and responsibilities of life after cancer.

## Cancer risk and responsibility

A second theme concerns the identification and management of the risk of cancer developing (73 articles), including the social and biographical context of cancer identification (17), the individualisation of risk and responsibility (28), and structural inequalities (28). While 12 articles considered a variety of cancer types, as in the previous section the majority of articles focused on women's experiences, with 44 on breast and/or cervical cancer risk, particularly in relation to the HPV vaccine. Twenty‐seven of these articles were on prevention or screening, with others focusing on areas such as media representations of cancer risk, and symptom identification. A smaller number (4) focused on men's experiences of prostate cancer. Within this section, we also incorporated literature considering genetic cancer risk – with all relevant articles here (5) focusing on the BRCA1/2 gene mutation.

### Biography, cancer risk and screening practices

Much of this research on the medical management of the risks of cancer took the individual lifeworld as a point of departure, reflecting the wider corpus of medical sociological literature in this field (Cox and McKellin 1999, Lupton and Tulloch 2002, Williams 2003). Focusing on how individuals experience and interpret cancer risk, prevention strategies and social location, researchers explored cancer (risk) in a variety of global settings, with a predominant focus on cervical and breast cancer screening (e.g. Lovell *et al*. 2007, Nguyen and Clark 2014, Team *et al*. 2013)

Deploying key medical sociology concepts such as ‘lay knowledge’ (Armstrong and Murphy 2008, Nekhlyudov *et al*. 2009) and lay models of illness causation (White *et al*. 2012), these authors explored how screening is conceptualised and understood in relation to identity, lifecourse and culture. For example, Armstrong and Murphy (2008) considered how UK women receiving information about cervical cancer screening deployed ‘lay understandings’ drawn from childbirth and menopause to make sense of risk. White *et al*. (2012) explored ‘lay models’ of illness causation among Zambian women considering cervical cancer risk, combining traditional medicine with health education messages. Authors have also sought to explain the avoidance of screening by attending to lay beliefs, for example Keeley *et al*. (2009) found that a fatalistic approach towards disease was mobilised by women as a way of reducing the stress associated with the uncertainties of cancer risks. Others explored how avoidance varied according to social differences, for example Thompson *et al*. (2012) described the ways in which masculinity intersected with other aspects of identity, such as ethnicity, to limit engagement with colorectal cancer screening.

Other researchers focused on delays in seeking a diagnosis for cancer, considering how individuals grappled with uncertainty about ‘what counts’ as a sign of illness (Andersen *et al*. 2015). These studies involved detailed analysis of interviewees’ accounts of the complexities of identifying sensations as symptoms (Andersen *et al*. 2010, Locock *et al*. 2016). Studies described how sensations deviating from expected bodily experiences were normalised by individuals when interpreted with reference to wider contextual factors such as the ageing process, menopause (Brandner *et al*. 2014), or personal stress (Andersen *et al*. 2010). Unger‐Saldana and Infante‐Castaneda (2011) also pointed to the role of fear in preventing women from seeking help for symptoms. In these studies, it was only when bodily sensations began to impair daily routines such as dressing, or when friends and family raised concerns (Unger‐Saldana and Infante‐Castaneda 2011), that apparently mundane sensations transformed into symptoms of illness (Brandner *et al*. 2014). Sociologists have also shown how biomedical technologies, such as those used in screening, play an important role in constituting symptoms by bringing the body into view (Blomberg *et al*. 2009). Others focused on how interactions with medical professionals could increase uncertainty about who was responsible for the identification of symptoms (Degeling *et al*. 2016). Together, this work demonstrates that a person's sense of being at risk of or diagnosed with cancer is not a simply a matter of their appreciation of biomedical indicators. Instead, this is a temporal, embodied and relational process, shaped by the cultural and institutional practices in which experiences of signs and/or symptoms are embedded.

### Individualising responsibility for risk

Another set of more critical literatures located these lay beliefs and practices in a wider analysis of citizenship and governmentality, within a broadly Foucauldian analytical framework, emphasising the relocation of responsibility for risk identification and management to the individual (Bunton and Petersen 2002; Clarke *et al*. 2003). Several of these articles concerned media representations of cancer risk. Abdelmutti and Hoffman‐Goetz (2009) found that fear and fright were common features of newspaper articles about cervical cancer, which may serve to heighten individual risk perceptions, though the extent of this differed within US and Canadian media. Musso and Wakefield's (2009) work also explores the portrayal of cancer risk in Canadian newspapers, and found that coverage most frequently emphasised the management of cancer risk through individual lifestyle change. Similarly, Chen *et al*. (2014) describe an emphasis on individual responsibilities for addressing cancer risk in media coverage of screening for breast cancer, noting that in the wake of debates surrounding the efficacy of breast cancer screening, its potential harms were less visible in the media than positive accounts from women or celebrities believing they had benefited from screening programmes.

Other researchers looked at negative media coverage of the HPV vaccine. Authors explored the sexual politics surrounding the vaccine, examining how conservative commentators' disapproval of the programme prevented some women from taking up the vaccine in the US (Casper and Carpenter 2008) and Australia (Rosenthal *et al*. 2007; see also Dyer 2010, Gullion 2011, Hilton *et al*. 2010). As these authors argued, women were presented as responsible for preventing cervical cancer via abstinence rather than participation in the programme, with men's role in the transmission of the virus disregarded.

Articles often foregrounded issues of responsibility. Armstrong (2007) drew on Foucault's work on governmentality, to demonstrate how the UK cervical screening programme portrayed all women as potentially at risk of cervical cancer, encouraging women to take responsibility for and act on threats to health by attending regular screening. Hill and Hayes (2015) applied similar analysis to public health initiatives and associated campaigns encouraging patient ‘awareness’ of symptoms of cancer, and Topping *et al*. (2013) explored how these practices locate the responsibility for late diagnosis with patients. A similar theme was taken up in other analyses of how cancer prevention approaches based around evidence‐based medicine (Bell and Ristovski‐Slijepcevic 2015), mathematical models to determine risk estimations for individuals (Holmberg and Parascandola 2010), or linking the risk of cancer to particular behaviours (Hooker *et al*. 2009) foreground individual responsibilities for risk management while giving ‘minimal attention to the social and environmental context needed to achieve this’ (Carter *et al*. 2009).

Another group of articles explored how potentially ‘at‐risk’ individuals (re)configured their accountability for cancer risk and its management. For some this involved intensified compliance, for example via participation in a clinical trial (Holmberg *et al*. 2015), or seeking out more frequent screening (Lindberg *et al*. 2013). Two articles explored how participants in screening spoke of ‘doing the right thing’, averting (later) costs to the health service in the UK (Chapple *et al*. 2008), or protect their health for their children's future (Lovell *et al*. 2007). This could also involve extending or reworking accountabilities, as in the case of women undergoing genetic testing for BRCA1/2 mutations, associated with a heightened cancer risk, who rationalised their participation as a means by which they could act to avoid illness, and continue to care and nurture others (Rowley 2007). These responsibilities were also found to extend to encouraging other women in the family to participate in screening, in the case of mothers encouraging uptake of HPV screening (Connell 2010).

Coping with the uncertainties of being at risk of cancer was another topic in this literature, particularly in relation to genetic risk. For example, in research with asymptomatic Israeli women testing positively for the BRCA1/2 mutation, Dagan and Goldblatt (2009) found that knowledge of genetic risk positioned participants within a ‘twilight zone’ between health and sickness, spurring their plans for risk management drawn in part from their mothers’ experiences of cancer. Two studies from Norway, emphasise how difficult it might be to engage in decision making, when medical knowledge of the meanings of genetic test results may be inconclusive, and a cautious framing of evidence is employed by medical professionals (Thomassen and Sarangi 2012). In the cases of uncertain genetic status, family history and past clinical experience may be drawn on more heavily than numerical (risk) information provided by genetic testing (Aasen and Skolbekken 2014). Research also suggests that these encounters, and the results of genetic testing, may be experienced as more uncertain for ethnic minority patients given the lack of ethnic diversity within research populations from which genetic data were derived (Butrick *et al*. 2014).

### Structural inequalities

Another body of literature identified for this review (28 articles) focused on structural inequalities in screening and prevention, identifying barriers faced by those from ethnic minority or socioeconomically disadvantaged groups in accessing interventions developed to manage individual cancer risk. This literature focused on minority groups within the UK, US and Australia, and low to middle income countries such as Brazil, taking both men's and women's experiences and a range of cancer types into account. Research in this area was focused on informing the design national screening programmes in order to improve participation by marginalised groups by identifying barriers to screening.

A range of barriers to ethnic minority communities were identified in these articles, including distrust of the medical system among African American (Allen *et al*. 2007, Wray *et al*. 2009) and Chinese (Gao *et al*. 2009) participants, misconceptions about the purpose of screening tests among Barbadian women, (Christian and Guell 2015), a lack of knowledge about national healthcare systems and screening (Arevian *et al*. 2011, Erwin *et al*. 2010, Kawar 2009, Team *et al*. 2013, Thorburn *et al*. 2013, Wangari Ngugi *et al*. 2012) and cultural and religious traditions (Lee *et al*. 2012, Wallace *et al*. 2014). Researchers emphasised the role of socioeconomic factors in preventing access to screening and preventive measures such as HPV vaccination (Patel *et al*. 2012, Paz‐Soldan *et al*. 2012, Schoenberg *et al*. 2010). In a French quantitative study, Bussière *et al*. (2016) highlight disparities in accessing cervical, breast and colorectal cancer screening, alongside GP and nurse care, between those with disabilities and the wider population. They found that these disparities were not due to cognitive or physical capabilities, but heavily influenced by socioeconomic factors which shaped individuals' participation in social activities. Other literature also focused on barriers embedded in the design of programmes, including language barriers to Hispanic and Southeast Asian women in the US (Griffiths and Udyavar 2011, Kue *et al*. 2014), and a lack of provision of breast and cervical screening and vaccine services to minority communities (Clevenger *et al*. 2012, Fernandez *et al*. 2009, Hutson *et al*. 2011, Souza de Bairros *et al*. 2011).

Although some of this literature tended towards a ‘deficit model’ of participants’ understanding, and sought to address this with improved information and education (Lee *et al*. 2012), other authors took a more critical approach, arguing that structural changes which addressed socio‐economic inequalities would be required to improve uptake, alongside information and choice, for example Bussière *et al*. (2016) advocate measures to increase social support and address socioeconomic disparities in order to improve participation in screening among those with disabilities (see also Jepson *et al*. 2007). The literature on gender as a barrier to screening also took a critical approach to the combination of social norms and mores which limit men's and women's engagement with certain kinds of screening, in a similar vein to the literature on biography and risk perceptions discussed previously. For example, Lu (2007) explores dominant depictions of masculinity and intimate relationships among African American men, and others consider the conventions of modesty or embarrassment as expressed by Vietnamese, Muslim and Malay women undergoing intimate examinations in the US (Dunn and Tan 2010, Faik Salman 2012, Nguyen and Clark 2014).

The negotiation of uncertainty via lay beliefs and practices, norms of citizenship and responsibilisation in the clinic and culture was a key theme in these articles. This work traces the experience of ‘being at risk’ of cancer across the spectrum of intimate and public life, exploring how individuals navigate risk from the embodied practices of interpreting ‘warning signs’, their responsibilities to kin and community and interactions with care‐givers. This has also contributed to calls for improvements in cancer screening and care and support strategies for affected families and the development of more appropriate screening infrastructures.

## Bioclinical collectives

Although the mainstay of sociological research on cancer over the last decade has focused on cancer experiences, including interactions and provision of cancer services for individuals at risk of, living with and beyond cancer, sociologists have also engaged with developments in oncology and cancer research as well as policy and regulation of cancer research and services. The focus of this corpus of literature is on professional practices and discourses, as well as patient involvement and governance, in the cancer field. This work explores the formations and social actions of scientific knowledge communities and technologies, drawing from the tradition of socio‐historical, laboratory and Actor Network Studies in STS (e.g. Fujimura 1996, Keating and Cambrosio 2012), and literature exploring the political economy and networks of cancer (e.g. Davis and Abraham 2013, Jain 2013, Stacey 1997).

We identified 53 articles which explored these topics across three sub‐themes: professional practices and relations (24), patients in research (10), and policy and regulation (19). Much of this work focuses on the ways in which cancer research and clinical teams, institutions and organisations in the developed world, particularly the USA and Europe, are coordinated, (e.g. Bourret *et al*. 2011), with only three articles considering low to middle income countries (Broom and Doron 2012 (India); Gibbon 2009; 2013 (Brazil and Cuba)).

### Professional practice

The articles on professional practices focused on how professionals work together, develop and implement new approaches and make decisions around diagnosis and treatment. Several authors explored the organisation of oncology, including historical studies on the processes of specialisation in gynaecological oncology (Zetka 2011) and the ways teams operated in relation to new initiatives such as quality improvement indicators (Gort *et al*. 2013). The influence of evidence‐based medicine on organisational arrangements, professional hierarchies and jurisdictions were also explored (Abel and Thompson 2011, Broom *et al*. 2009). Together these studies show the complexity of professional approaches, organisational tools and processes deployed to improve cancer treatments and diagnosis in this period, but they do not suggest that any particular strategy such as specialisation or standardisation were easily achieved or especially influential. Rather they tell the story of increasingly complex, dynamic networks of tools, categories and jurisdictions of expertise which have been brought to bear on cancer. For example, in explorations of how diagnosis works, authors have detailed various kinds of expertise and negotiations which are involved in the daily work of the clinic, including the negotiations that take place in relation to validity and reliability of data and expertise (Gross 2009), and the materialisation of ‘good’ and ‘bad’ cancers as they relate to professional identities and jurisdiction (Kazimierczak and Skea 2015). Exploring regulatory discussions about the impact on genomics on the diagnostic process, Bourret *et al*. (2011) highlight how the locus of clinical decision making is shifting to take account of this new information. Gibbon (2009) also considers how genomics and personalised medicine are differentially embedded in high and low income national contexts and public health cultures. Others focus on the developing research‐clinical nexus, including how interdisciplinarity works in research contexts (Centellas *et al*. 2014) and transnational arrangements of cancer genetics (Gibbon 2013). The ways in which the boundaries between research and clinical practice are increasingly blurred in the context of genomic medicine is also explored, as clinical trials increasingly take on an experimental ethos (Nelson *et al*. 2014). Professionals’ and patients’ navigation of these new molecular regimes involve ambivalence and multiple interpretations. Prior (2007) explores how the gene becomes a ‘boundary object’ supporting these different positions and Darling *et al*. (2016: 51) consider how professionals make meaning out of molecularisation through, ‘epistemic hinges to facilitate a turn from efforts to understand social and environmental exposures outside the body, to quantifying their effects inside the body’.

### Patient and public participation

Professionals have become increasingly engaged with publics through various processes of participation over the period covered by the review, and this is reflected in a range of articles on this topic. Exploring the tensions that can arise with respect to who is involved and how (Martin 2008, Martin and Finn 2011), researchers have detailed the problems and possibilities of these new arrangements. Sinding *et al*. (2012) raised concerns about the exacerbation of inequalities in patient involvement which are often obscured as a result of inattention to the resources required to be a participant, and Martin and Finn (2011) considered the challenges in bringing patients into already heterogeneous teams of cancer professionals. The different approaches and relations of patients and publics involved in cancer services and research were also explored, from the supportive charity whose activities assist with enrolment of patients (Ashmore 2012) to the critical activist community advocating for alternative approaches (Dawes 2012) and the expert involved patients who are becoming ‘professionalised’ (Thompson *et al*. 2012).

Other authors have looked closely at patients’ various roles in the burgeoning field of cancer research, picking up this theme of the blurred boundaries between treatment and research, to consider how patients take an active role in constituting the research agenda and environment (Armstrong and Morris 2010, McIntyre *et al*. 2013, Morris *et al*. 2009). Problems with exclusion from clinical trials, specifically barriers to participation experienced by minority groups, have also been explored (Robinson and Trochim 2007), as has the role of community health workers in tackling this (Schutt *et al*. 2010). Drawing on ethnography and post‐structuralist theories, some scholars take a more critical stance, exploring the replication of social inequalities in research infrastructures which draw participants who have better access to medical facilities (Joseph and Dohan 2012). This challenges the lack of regard for participants’ lived experiences, particularly evident in paradoxes that arise from the removal of individual particulars from death statistics, ‘ghosting’ their lives (Jain 2010). The informed consent process is also a focus of critical analysis, including the ways in which it is used to manage the conflicts of interest in research and clinical care in cancer genetics clinics (Hallowell *et al*. 2009), and the dangers of its operation as a ‘technical fix’ which does not capture the complexities of how patients make decisions about trial or other research participation in practice (Armstrong *et al*. 2012, Vermeulen *et al*. 2011).

### Policy and regulation

Various authors explored the process of policy formation for interventions such as bowel screening programmes (Flitcroft *et al*. 2011) and the HPV vaccine (Mishra and Graham 2012, Paul 2016) and the kinds of imagined recipients, evidence, storylines, influence and expertise brought to bear in the structuring of provision. This included articles on evidence‐based medicine in cancer service development, using a frame of governmentality (Ferlie *et al*. 2012). Others challenged overly‐simplistic understandings, for example of vaccine choice (Mishra and Graham 2012), and more applied research sought to build communication strategies and resolve differences in understandings of environmental risk for breast cancer (Potts *et al*. 2007). Patient participation in policy‐making was also explored by several authors, including the influence of patient advocates on drug reimbursement (Nahuis and Boon 2011), opposition to policy recommendations (Barker and Galardi 2011, Lavariega Monforti and Cramer 2014), and the role of pharmaceutical companies in encouraging certain kinds of advocacy to build awareness and markets for their products (Gottlieb 2013).

Echoing the themes in research on professional organisation and sense making, other authors explored the influence of regulatory processes and guidelines on clinical trials, particularly processes concerned with standardisation (Keating and Cambrosio 2009), stabilisation and the production of evidence (Knaapen *et al*. 2010). The ways in which regulatory actors respond to the plethora of new interventions in the cancer field was also a focus of analysis, including the processes through which approval is accelerated in response to pharmaceutical industry interests, rather than patient advocacy as is typically claimed (Davis and Abraham 2011, see also Abraham and Ballinger 2012). This literature also considered how expectations around new interventions such as genomic risk profiling are managed (Faulkner 2012), and the influence of the media as well as pharmaceutical industry representatives and consumer groups (Gabe *et al*. 2012). As Hogarth *et al*. (2012) argue, these processes of decision making are led by commercial interests but change is incremental rather than revolutionary.

These studies have explored scientific and regulatory activities and organisations around cancer, including professional responsibilities for data and new kinds of interdisciplinarity. These developments have evolved alongside and in relation to particular kinds of patient experiences, responsibilities and identities, not least the obligation to participate in trials and donate material for further research as part of being an active and informed patient. Sociologists have explored how these processes evolve and the reasons behind this, as well as advocating for and perhaps enabling greater, meaningful involvement of patients and publics therein.

## Conclusion

Sociologists have traced and interrogated a plethora of experiences, arrangements and responsibilities in relation to cancer over the last decade. This has included patient, professional and regulatory engagement with tests and treatments, including genetic and genomic. It has also gone beyond this to explore a broader range of social and technical transformations in identities, organisation and jurisdictions. Three noteworthy conclusions can be drawn.

First, much of the sociological research in this period has pointed to the complex processes through which new kinds of biomedical knowledge are shaped by and shape people's identities and organisational practices. However, sociological research does not suggest that biomedical and specifically genomic developments in this period are driving the major transformation that some of the highly optimistic narratives in this field suggest (Hedgecoe 2004), nor are they responsible for a dramatic paradigm change in cancer services or care. Instead sociological research suggests that biomedical knowledge has, in many cases, introduced new kinds of uncertainties, work and responsibilities to be navigated by cancer patients and professionals. This includes the reworking of accountabilities and conceptualisations of cancer related to individuals’ lifeworlds and cultures (e.g. Armstrong 2007), as well as processes of alienation, exclusion and barriers to access (e.g. Broom and Doran 2009; Robinson and Trochim 2007). New kinds of biomedical innovations and understandings of cancer are therefore best understood as part of wider processes of change rather than drivers of transformation in already complex social arrangements and identities around cancer, including patients’ sense of embodied risk, relationships between and among patients and professionals and new regulatory and organisational infrastructures.

Second, our review points to some important social as well as biomedical innovations that have occurred over this period, notably in the identities and the organisation of cancer research and care. These developments are important in shaping experiences of being at risk of, living with and beyond cancer, as well as professionals’ experiences of researching and caring for those affected and, as such, can be considered as innovations in their own right. The generation of a diverse and lively set of identity narratives about living at risk of, with and/or beyond cancer is one such development. These identities, developed and proliferated by a range of activist and support communities, as well as through sociological research, challenge stereotypical narratives and ideals of heroism, battles and war and becoming a better person due to cancer (Burke *et al*. 2012, Kaiser 2008). Other changes have arguably taken place in the forms of care that are available to people affected by cancer, including a more nuanced appreciation of how care providers can listen to and support patients differently (Kue *et al*. 2014, Sinding *et al*. 2010). Sociological explorations of cancer research, clinical and regulatory processes, have highlighted a range of innovations in the organisation of cancer services, notably in the paradigms and biomedical platforms of clinical trials (Keating and Cambrosio 2009, Kohli‐Laven *et al*. 2011), evidence‐based medicine and patient involvement mechanisms (Martin *et al*. 2011, J. Thompson *et al*. 2012).

Third, our review has revealed the role of sociologists and fellow social researchers in these processes. Sociology has documented and explored these developments but has also participated in them. We have found that sociologists and fellow social researchers sometimes replicate dominant processes and approaches in their own work, for example via a focus on particular cancers or communities, notably breast cancer and white women in high‐income countries, to the neglect of others, notably other common cancers, such as lung cancer, and marginalised groups. However, sociologists and colleagues in cognate fields have also documented and in some cases been involved in the development of counter‐identities which reject dominant narratives in favour of more critical and complex understandings of what it means to live with the uncertainties involved with being at risk of, with or beyond cancer (e.g. Steinberg 2015). This has included critical interventions in sociological theories and frameworks such as biographical disruption as well as dominant narratives and processes such as ‘pink‐washing’ of cancer (Cobb and Starr 2012, DeShazer 2012). Sociologists have also been part of a general trend to take better account of the experiences of, and to seek to better involve, patients and publics in cancer innovation, policy and regulation. They have contributed to a growing awareness of the work involved in being at risk of or living with and beyond cancer, including the unintended consequences of greater patient and public involvement which can intensify responsibilisation of particular individuals at the same time as it marginalises others.

This review also suggests some important future directions for sociological research on cancer. The humanistic ethos of sociological work on the experiences, identities and responsibilities of cancer is a welcome antidote to more remote analyses of technological or regulatory processes. Yet, sociological analysis of research collectives are also vitally important for our understanding of broad shifts in the constitution of cancer as a disease entity. There is now a need to continue to link and move beyond these approaches to engage with wider sociotechnical processes of involvement, identification and distribution of power and status in the cancer field – across research, treatment and cultural realms. We will only be able to present a comprehensive account of cancer by situating patient and public experiences of risk, responsibility, and participation in research, treatments and surveillance, in relation to the technologies, science and expertise through which they are produced. This will provide the evidence and analytical tools that may help support individuals to navigate and successfully influence research and service provision beyond the limited scope offered by the identity politics of consumer society.
